# Clinical Subgroups and Treatment Outcomes in Idiopathic Normal Pressure Hydrocephalus: Application of Machine Learning-Based Clustering

**DOI:** 10.1227/neuprac.0000000000000257

**Published:** 2026-06-11

**Authors:** Emalee J. Burrows, Linda D'Antona, James Booker, Shahriar Islam, Faraaz Shariff, Deehan Matthew, Michelangelo Monticelli, Haris Rehman, Hani J. Marcus, Ahmed K. Toma

**Affiliations:** 1 UCL Queen Square Institute of Neurology, London, UK;; 2 Victor Horsley Department of Neurosurgery, The National Hospital for Neurology and Neurosurgery, University College London NHS Foundation Trust, London, UK;; 3 The Royal London Hospital, Barts Health NHS Trust, London, UK

**Keywords:** Artificial intelligence, Clinical phenotypes, Clustering, DESH, Idiopathic normal pressure hydrocephalus, Unsupervised machine learning, Ventriculoperitoneal shunt

## Abstract

**BACKGROUND AND OBJECTIVES:**

The variability in clinical phenotype, radiological features and treatment outcomes of idiopathic normal pressure hydrocephalus (iNPH) poses significant challenges for diagnosis and management. This study aimed to use artificial intelligence techniques, specifically unsupervised machine-learning clustering, to identify meaningful clinical subgroups and outcome trajectories after ventriculoperitoneal shunt insertion.

**METHODS:**

In this retrospective single-center case series, clustering methods were applied to characterize patients with shunt-responsive iNPH, based on demographic, clinical, and radiological variables. Clinical data included preoperative symptoms and postoperative shunt response and long-term outcomes. All radiological data were extracted by 2 senior neuro-radiologists blinded to the clinical data.

**RESULTS:**

A total of 187 patients were identified, with a mean age of 75.6 ± 6.6 years, and comprising 69 (37%) women. Patients were followed up for a median duration of 28 months [IQR, 16-59]. Ward's hierarchical agglomerative clustering identified 4 clearly defined subgroups with distinct clinical outcome trajectories. Individuals with probable iNPH typically aligned with one of the following profiles: (1) disproportionately enlarged subarachnoid space hydrocephalus-positive, (2) dilated sylvian fissure hydrocephalus, (3) small-vessel disease with ventriculomegaly, and (4) marked ventriculomegaly.

**CONCLUSION:**

These findings challenge the traditional view that iNPH is a single disease and provide new insights into its pathophysiology. While disproportionately enlarged subarachnoid space hydrocephalus remains a valuable indicator of shunt responsiveness, its low negative predictive value highlights the importance of considering additional iNPH phenotypes. Incorporating cluster-based phenotyping into clinical pathways may improve prognostic prediction, enhance patient counselling before shunt surgery, and enable more targeted management strategies. Attention to such subgroups should be considered in future iNPH research.

**ABBREVIATIONS:**
DESH: disproportionately enlarged subarachnoid space hydrocephalus
DSF: dilated sylvian fissures
EI: Evans Index
iNPH: idiopathic normal pressure hydrocephalus
NPV: negative predictive value
SDH: subdural haematoma
THC: tight high convexity.

Idiopathic normal pressure hydrocephalus (iNPH) is a condition characterized by gait disturbance, cognitive impairment, and urinary incontinence in older adults.^1^ Despite well-defined diagnostic frameworks outlined in international guidelines,^2^ substantial variability exists in symptoms, imaging, treatment response, and prognosis. This variability complicates stratification of patients for shunt insertion, which remains the mainstay of treatment.^3^

Notably, as many as half of patients with a clinical presentation consistent with iNPH fail to exhibit a positive response to shunt surgery.^1^ At present, no clinical^4^ or radiological marker^5^ reliably identifies which patients will definitely improve following shunting using traditional statistical methods.^1^ Although gold standard diagnostic procedures such as the lumbar tap test demonstrate a favorable positive predictive value (PPV) of 92%, it is limited in excluding nonresponders with a negative predictive value (NPV) of 37%.^6^ Growing evidence suggests that iNPH patients may fall into distinct clinical subtypes.^7,8^ These subgroups, while presenting similarly, may have variable long-term outcomes and differential responses to shunting and shunt valve settings.

Unsupervised machine-learning techniques, such as clustering analysis, provide a data-driven approach for identifying naturally occurring subgroups without relying on predefined classifications or relationships. This project aims to analyze and categorize these patient subgroups to better characterize their clinical presentations, radiological features, and outcome trajectories. By characterizing these phenotypes, we aim to offer a novel approach to improving patient selection for surgery and to inform more personalized treatment strategies, enabling patient-centered counselling, optimized shunt programming workflows, and long-term follow-up tailored to each individual's profile.

## METHODS

### Study Population

This was a retrospective clinical study conducted at the National Hospital for Neurology and Neurosurgery, approved as a service evaluation by the local clinical governance committee, and reported according to Strengthening the Reporting of Observational Studies in Epidemiology guidelines.^9^ As a retrospective service evaluation using routinely collected clinical data, informed consent was not required. Patients were eligible if they were probable iNPH defined in International iNPH Guidelines,^10^ had received a ventriculoperitoneal shunt, had a minimum follow-up of 6 months, and had preoperative brain imaging. Patients with secondary were iNPH excluded, and data extracted from electronic healthcare records from November 22, 2013, to November 22, 2023.

### Clinical Evaluation

Demographic variables, medical history, and presenting symptoms were extracted. Symptoms were evaluated at baseline, 6 months after shunting, and most recent follow-up. A positive response was defined as a clinically significant improvement reported by the hydrocephalus consultant, registrar, or dedicated specialist hydrocephalus nurse. The duration of response was from the time of shunt insertion until the time at which a clinically significant improvement from baseline was no longer observed. This was considered both globally and for each symptom within the iNPH triad (gait disturbance, cognitive impairment, and urinary incontinence).^11^ Measurement tools included the 10-m walk test and Wechsler Adult Intelligence Scale, with ≥10% improvement in time, stride length, verbal intelligence quotient, or performance intelligence quotient considered clinically significant.^12^ Deterioration was recorded and defined as a decline in symptoms compared with baseline, alongside quality of life assessed using the modified Rankin Scale.

### Radiological Investigations

The presence of disproportionately enlarged subarachnoid space hydrocephalus (DESH) was defined according to the Third Edition of the Japanese Guidelines for Management of iNPH requiring enlarged ventricles (Evans Index [EI] >0.3), tight high convexity (THC), and dilated sylvian fissures (DSF).^3^ Two senior neuro-radiologists (S.I., F.S.) extracted imaging features and were blinded to outcomes.

The Fazekas scale was used for characterization of radiologically evident vascular disease, grading both periventricular white matter and deep white matter changes.^13^ This scale has been histopathologically validated,^14^ enables quantitative analysis with good inter-rater reliability,^15^ and demonstrated reliability on MRI^16^ and computed tomography.^17^ The callosal angle, measured in the coronal plane at the posterior commissure, and skull dimensions were also included.

### Shunt-Related Outcomes

Shunt outcomes included number of valve-setting changes, optimal setting, setting at most recent follow-up, and the presence of complications such as subdural hematoma (SDH) or shunt failure requiring revision. The time taken to reach the optimal setting was also recorded. Within our unit, patients are proactively followed up to adjust valve settings and facilitate titration for optimal symptom improvement. Optimal setting was defined when the patient's walking speed, or symptom control was optimal, without features of overdrainage or SDH.

### Statistical Analysis

Cluster analysis was performed using demographic, baseline clinical, radiological, and outcome data. Gower's^18^ dissimilarity index was used to measure similarity and dissimilarity between patients. Duda-Hart pseudo-T2 and Calinski-Harabasz pseudo F indices were assessed to determine the optimal number of clusters. Clustering results were visualized using a dendrogram and radar plot. All data were assessed for normality and statistical methods adjusted accordingly. Post hoc comparisons between clusters included analysis of variance or Kruskal-Wallis tests for continuous variables and χ^2^ tests for categorical variables. Bonferroni correction was applied for multiple comparisons. Shunt response was assessed evaluating improvement, stability of symptoms, and deterioration using the Kruskal-Wallis test. Significance level was α = 0.05 and tests two-tailed. A multivariate regression was also performed to assess significant predictors of shunt responsiveness; McFadden's R^2^ was used to assess model fit. Statistical analyses were performed using R (version 3.2.4, R Foundation) and STATA (version 18.0).

## RESULTS

### Study Participants

Overall, 187 patients were included from 372 screened, all with complete data. Baseline characteristics are presented in Table 1. The mean age of the patients was 75.6 ± 6.6 years, comprising 69 (37%) women. A total of 44 (24%) reported smoking and 157 (84%) at least one vascular risk factor. The most common symptom was gait disturbance 187 (100%), followed by cognitive deficits 158 (84%) and urinary symptoms 142 (76%). Fifty-four patients (29%) were DESH-positive, with 70 (37%) having features of THC and 100 (53%) DSF. The mean callosal angle was 71.2 ± 20.2, with an average Fazekas score of 3.24 ± 1.81. Patients were followed for a median duration of 28 months [16-59].

**TABLE 1.** Demographic, Clinical, and Radiological Characteristics of the Study Population

Characteristic	Value
Age at shunt insertion (y), mean ± SD	75.6 ± 6.6
Women/men, n	69/118
Clinical features, n (%)	
Gait	187 (100)
Cognitive deficit	158 (84)
Urinary symptoms	142 (76)
Headaches	24 (13)
Visual symptoms	1 (1)
Seizures	6 (3)
Syncope or presyncope	5 (3)
Dysphagia	1 (1)
Speech abnormalities	8 (4)
Pulsatile tinnitus	1 (1)
PMH, n (%)	3 (4)
Alcohol (moderate to heavy consumption)	34 (13)
Smoker	44 (24)
CVA	51 (27)
CAD	17 (9)
Hyperlipidemia	46 (25)
Diabetes	40 (21)
Hypertension	91 (49)
OSA	5 (3)
Radiological, n (%)	
DESH positive	54 (29)
THC	70 (37)
DSF	100 (53)
Fazekas total, mean ± SD	3.24 ± 1.81
Fazekas PVWM, mean ± SD	1.8 ± 1.0
Fazekas DWM, mean ± SD	1.4 ± 1.0
EI, mean ± SD	0.38 ± 0.04
Callosal Angle, mean ± SD	71.2 ± 20.2

CAD, coronary artery disease; CVA, cerebrovascular accident; DESH, disproportionately enlarged subarachnoid space hydrocephalus; DSF, dilated sylvian fissure; DWM, deep white matter; EI, Evans-Index; OSA, obstructive sleep apnea; PMH, past medical history; PVWM, periventricular white matter; THC, tight high convexity.

### Cluster Analysis

**FIGURE 1.**
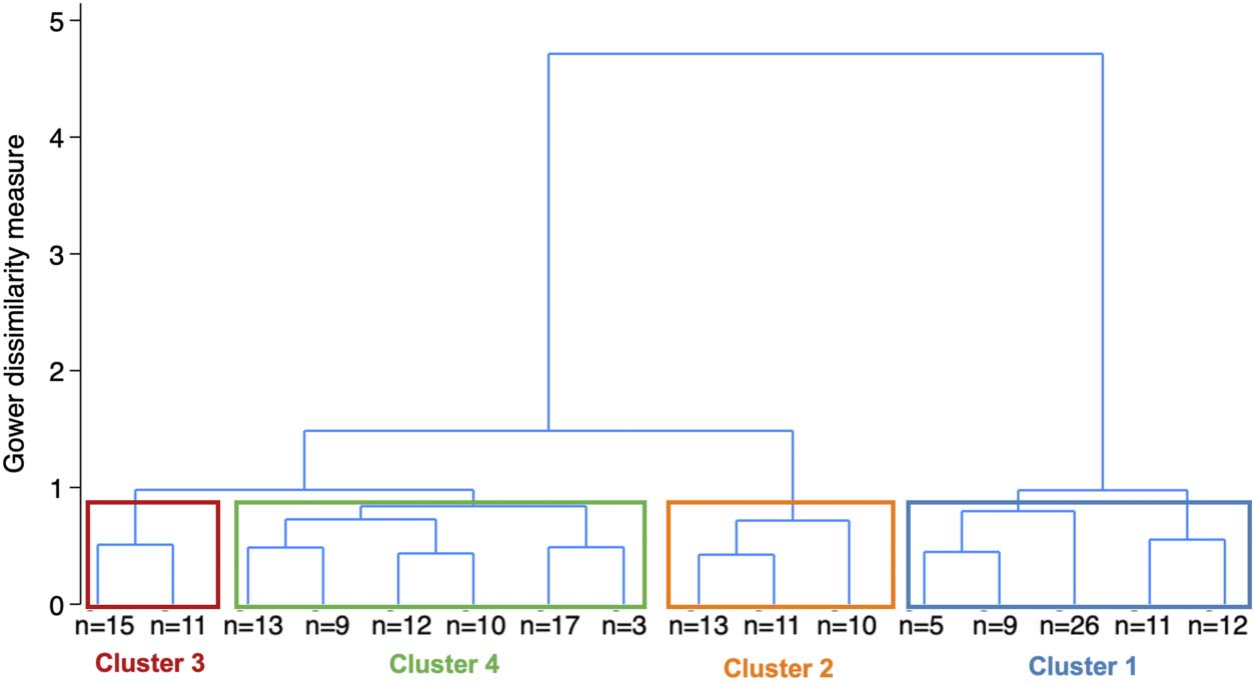
Dendrogram showing 3 main clusters and a fourth highly dissimilar group (cluster 1). The horizontal axis represents individual patients, and the vertical axis provides a measure of the dissimilarity between each of the cluster groupings.

**FIGURE 2.**
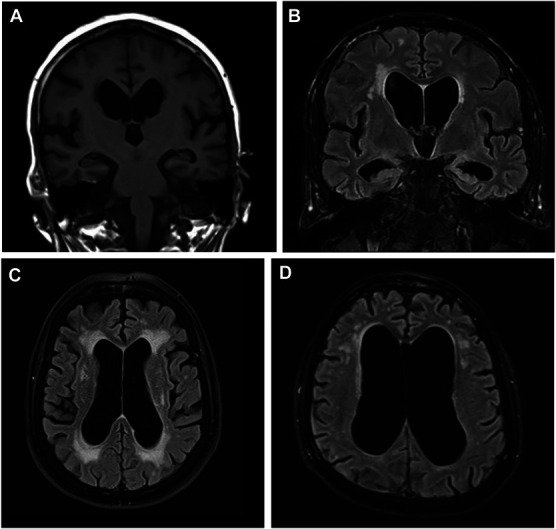
Imaging demonstrating typical findings for each of the clusters. **A**, Disproportionately enlarged subarachnoid space hydrocephalus-positive, **B**, dilated sylvian fissure hydrocephalus, **C**, small vessel disease with ventriculomegaly, and **D**, marked ventriculomegaly.

Cluster separation was performed using Ward's hierarchical agglomerative clustering, categorizing patients into 3 distinct groups with a fourth highly dissimilar cluster (Figure 1). Key aspects defining groups were age, gender, gait and urinary symptom duration, smoking status, comorbidities, DESH, EI, and Fazekas score. Key features are summarized in Table 2 with example imaging in Figure 2.

**TABLE 2.** Summary Statistics of Variables for the Main Clusters Observed

Variables	Cluster 1 (n = 63)	Cluster 2 (n = 34)	Cluster 3 (n = 26)	Cluster 4 (n = 64)	*P*-value
Women/men, n	27/36	1/33	21/5	20/44	**.000**
Age (y), mean ± SD	76.6 ± 6.0	76.9 ± 6.3	78.3 ± 7.0	73.0 ± 6.4	**.0005**
Baseline mRS, mean ± SD	3.03 ± 0.86	2.79 ± 0.77	2.96 ± 0.96	2.92 ± 0.70	**.5782**
Clinical features, n (%)					
Gait	63 (100)	34 (100)	26 (100)	64 (100)	1.0
Cognition	52 (83)	28 (82)	24 (92)	54 (84)	.679
Urinary	44 (70)	22 (65)	21 (81)	55 (86)	.059
No. of symptoms in iNPH triad, 1/2/3	7 (11)	5 (15)	0	0	**.044**
	19 (30)	8 (24)	7 (27)	19 (30)	
	37 (59)	21 (62)	19 (73)	45 (70)	
Headache	4 (6)	2 (6)	7 (27)	11 (17)	**.023**
Visual symptoms	0	0	1 (4)	0	.101
Seizures	1 (2)	0	3 (12)	2 (3)	.058
Syncope or presyncope	2 (3)	1 (3)	0	2 (3)	.841
Dysphagia	3 (5)	2 (6)	0	3 (5)	.587
Speech abnormality	0	0	0	1 (2)	.697
Pulsatile tinnitus	1 (2)	0	0	0	**.577**
Symptom duration (mo), median [IQR]	24 [12-36]	24 [12-36]	24 [14-36]	24 [18-48]	.0578
Gait symptom duration (mo), median [IQR]	18 [12-36]	24 [12-24]	24 [14-36]	24 [18-48]	**.0053**
Cognitive symptom duration (mo), median [IQR]	12 [0-24]	12 [0-24]	21 [3-36]	12 [4.5-36]	.4690
Urinary symptom duration (mo), median [IQR]	12 [0-24]	10.5 [0-24]	21 [6-24]	24 [12-36]	**.0065**
PMH, n (%)					
Alcohol (moderate to heavy consumption)	12 (19)	11 (32)	2 (8)	9 (14)	.145
Smoker	12 (19)	8 (24)	1 (4)	23 (36)	**.019**
CVA	21 (33)	11 (32)	5 (19)	14 (22)	.334
CAD	6 (10)	5 (15)	2 (8)	4 (6)	**.573**
Hyperlipidemia	19 (30)	8 (24)	6 (23)	13 (20)	.629
Diabetes	12 (19)	10 (29)	6 (23)	12 (19)	.611
Hypertension	38 (60)	20 (59)	10 (38)	23 (36)	**.018**
OSA	1 (2)	0	0	4 (6)	.166
No. of comorbidities, mean ± SD	1.31 ± 1.01	1.44 ± 1.31	1.04 ± 0.91	0.97 ± 0.99	.1058
Radiological					
DESH	54 (86)	0	0	0	**.000**
THC	62 (98)	1 (3)	1 (4)	6 (9)	**.000**
DSF	57 (90)	31 (91)	7 (27)	5 (8)	**.000**
Fazekas score, mean ± SD	3.83 ± 1.74	2.53 ± 1.31	3.38 ± 1.92	2.97 ± 1.91	**.0033**
PVWM score, mean ± SD	2.21 ± 0.83	1.53 ± 0.75	1.88 ± 0.99	1.58 ± 1.10	**.0006**
DWM score, mean ± SD	1.62 ± 1.05	1.00 ± 0.69	1.50 ± 1.07	1.39 ± 0.99	**.0303**
EI, mean ± SD	0.38 ± 0.04	0.37 ± 0.03	0.37 ± 0.06	0.39 ± 0.05	**.0185**
Skull dimensions (mm), mean ± SD	130.5 ± 6.10	136.1 ± 6.94	131.0 ± 5.74	135.5 ± 6.75	**.0000**
Callosal angle, mean ± SD	63.13 ± 13.42	75.54 ± 22.92	82.84 ± 25.52	72.22 ± 18.97	**.0001**

CAD, coronary artery disease; CVA, cerebrovascular accident; DESH, disproportionately enlarged subarachnoid space hydrocephalus; DSF, dilated sylvian fissure; DWM, deep white matter; EI, Evans-Index; iNPH, idiopathic normal pressure hydrocephalus; mRS, modified Rankin Scale; OSA, obstructive sleep apnea; PMH, past medical history; PVWM, periventricular white matter; THC, tight high convexity.

Boldface type indicates statistical significance.

#### Cluster 1: DESH-Positive

Cluster 1 was a distinct group of DESH-positive patients exhibiting hyperacute callosal angles. This group presented with a short duration of gait (median 18 months [12-36]) and urinary disturbance (median 12 months [0-24]), fewer additional symptoms, and often a history of hypertension (60%). They required the fewest shunt adjustments (median 1 adjustment [0-2]) alongside cluster 2 (median 1 adjustment [0-3]), with 76% demonstrating improvement in gait, 24% in cognitive symptoms, and 21% in urinary symptoms at 6 months. Overall, 78% experienced clinically significant improvement, sustained for a median of 21 months [9-44] of the follow-up period of 33 months [17-64].

#### Cluster 2: Dilated Sylvian Fissure Hydrocephalus

Cluster 2 were almost exclusively male (97%) and presented with fewer symptoms of the iNPH triad, with the lowest rate of urinary symptoms. They had the lowest vascular burden on imaging and often demonstrated DSF (91%) without THC (3%). These patients showed favorable improvement from shunting (79%) with none experiencing deterioration at 6 months. Improvement lasted a median of 20 months [3-28] of the follow-up period of 22.5 months [16-28].

#### Cluster 3: Small Vessel Disease with Ventriculomegaly

Patients in this subgroup were older (mean age 78.3 ± 7.0) and often female (n = 21, 86%).

They presented with more symptoms of the iNPH triad as well as headaches (27%), preexisting seizures (12%), and visual changes (4%). Imaging demonstrated the second highest vascular burden relative to other clusters, as reflected by Fazekas scoring and the broadest callosal angles, without the typical DESH pattern seen in cluster 1. These patients required more valve adjustments and higher valve setting to avoid complications. Although they exhibited an initial response to shunting (median duration 15 months [7-30] of the follow-up period of 24.5 months [14-53]), they were prone to progressive decline in gait and cognitive function, with 35% demonstrating improvement at 6 months.

#### Cluster 4: Marked Ventriculomegaly

Cluster 4 were the youngest (mean age 73.0 ± 6.4) and presented with more triad symptoms, in addition to headaches (17%) and preexisting seizures (3%). They were characterized by the largest ventricles (EI 0.39 ± 0.05) and enlarged skull dimensions (mean 135.47 ± 6.75 mm). These patients demonstrated the best overall response at 6 months (84%), with similar improvements in quality of life (modified Rankin Scale change −0.39 ± 0.79), although required more shunt adjustments. They experienced the longest improvement of 24 months [15-51] of the follow-up period of 34 months [18-74.5].

### Outcome and Shunt Details

**FIGURE 3.**
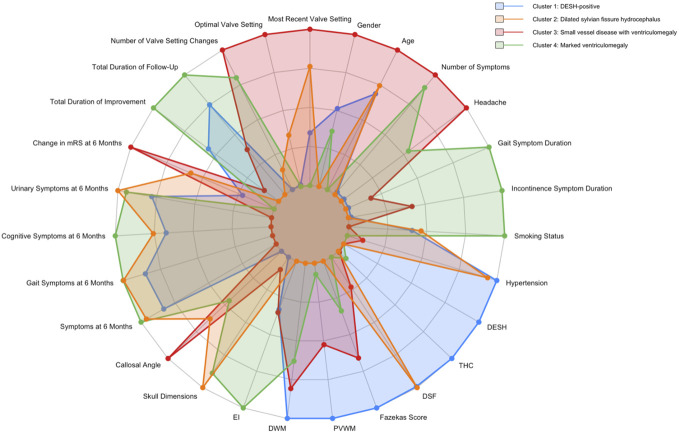
Radar plot of the standardized cluster means illustrating the distinct clinical, radiological, and outcome profiles for each of the 4 clusters where features were identified as statistically significant (*P* < .05). This visualization highlights the multidimensional differences between the various idiopathic normal pressure hydrocephalus subtypes. DESH, disproportionately enlarged subarachnoid space hydrocephalus; DSF, dilated sylvian fissure; DWM, deep white matter; EI, Evans-Index; mRS, modified Rankin Scale; PVWM, periventricular white matter; THC, tight high convexity.

Figure 3 and Table 3 summarize outcomes and shunt details for each cluster. Shunt insertion resulted in improvement in 75% of patients. Radiologically evident SDH occurred in 15%, often following valve adjustment aimed at deriving further benefit. 3% required shunt revision, with no significant differences between clusters. Patients demonstrated a clinically significant improvement for a median of 21 months [11-40] of the follow-up period of 28 months [16-59], taking a median of 1 month [0-12] to reach optimal symptom control. The multivariate regression model demonstrated a modest explanatory power (McFadden pseudo R^2^ = 0.15) and did not reach statistical significance (*P* = .079), supporting the hypothesis that linear modelling may insufficiently capture the multidimensional heterogeneity of this patient cohort.

**TABLE 3.** Outcomes and Shunt Details Presented per Cluster at the Time of Follow-up

Variables	Cluster 1 (n = 63)	Cluster 2 (n = 34)	Cluster 3 (n = 26)	Cluster 4 (n = 64)	Total (n = 187)	*P*-value
Complication—subdural hematoma, n (%)	9 (14)	6 (18)	5 (19)	8 (13)	28 (15)	.827
Complication—shunt revision, n (%)	1 (2)	2 (6)	1 (4)	1 (2)	5 (3)	.558
Symptoms at 6 mo, improved/stable/deteriorated, n (%)	49 (78)	27 (79)	9 (35)	56 (88)	141 (75)	**.000**
	8 (13)	7 (21)	7 (27)	5 (8)	27 (14)	
	6 (10)	0	10 (38)	3 (5)	19 (10)	
Gait symptoms at 6 mo, improved/stable/deteriorated, n (%)	48 (76)	27 (79)	8 (31)	54 (84)	137 (73)	**.000**
	9 (14)	7 (21)	8 (31)	7 (11)	31 (17)	
	6 (10)	0	10 (38)	3 (5)	19 (10)	
Cognitive symptoms at 6 mo, improved/stable/deteriorated, n (%)	15 (24)	9 (26)	1 (4)	28 (44)	53 (28)	**.000**
	46 (73)	25 (74)	17 (65)	36 (56)	124 (66)	
	2 (3)	0	8 (31)	0	10 (5)	
Urinary symptoms at 6 mo, improved/stable/deteriorated, n (%)	13 (21)	9 (24)	2 (8)	16 (25)	40 (21)	**.015**
	50 (79)	25 (74)	22 (84)	48 (75)	145 (78)	
	0	0	2 (8)	0	2 (1)	
Change in mRS at 6 mo, mean ± SD	−0.25 ± 0.57	−0.03 ± 0.90	0.23 ± 0.76	−0.39 ± 0.79	−0.19 ± 0.77	**.0022**
Total duration of improvement (mo), median [IQR]	21 [9-44]	20 [3-28]	15 [7-30]	24 [15-51]	21 [11-40]	**.0424**
Total duration of follow-up (mo), median [IQR]	33 [17-64]	22.5 [16-28]	24.5 [14-53]	34 [18-74.5]	28 [16-59]	**.0266**
No. of valve setting changes, median [IQR]	1 [0-2]	1 [0-3]	3 [1-3]	2 [1-3]	2 [1-3]	**.0133**
Optimal valve setting (cmH2O), mean ± SD	4.6 ± 1.8	5.3 ± 3.9	6.7 ± 5.3	4.6 ± 2.8	5 ± 3.3	**.0207**
Time to optimal setting (mo), median [IQR]	0 [0-12]	1 [0-8]	3.5 [0-11]	4 [0-16]	1 [0-12]	.6683
Valve setting at most recent follow-up (cmH2O), mean ± SD	4.6 ± 3.5	5.8 ± 4.4	6.4 ± 3.8	3.8 ± 2.8	4.8 ± 3.6	**.005**

mRS, modified Rankin Scale.

Bold values indicate statistical significance (*P* < .05).

## DISCUSSION

### Principal Findings

Patients typically presented with symptoms and radiological features consistent with probable iNPH, outlined in international guidelines.^10^ Despite this well-defined diagnostic framework, clinical heterogeneity remains a hallmark of iNPH. The median duration of improvement of 21 months of the follow-up period of 28 months highlights the heterogenous response of the iNPH patient cohort to shunt surgery. Some demonstrate long term improvements, others maintain baseline function status after shunting and some deteriorate despite valve setting optimization, thus emphasizing the need for assessing individual patient phenotypes to guide management. Within our cohort, patients with probable iNPH separated into distinct clinical and radiological clusters, each demonstrating characteristic demographics, comorbidities, symptom profiles, imaging patterns, valve setting requirements, and outcome trajectories.

### Cluster 1: DESH-Positive

Comprising 33.9% (n = 63) of patients, cluster 1 was highly distinct. They represented predominantly DESH-positive patients with characteristic hyperacute callosal angle, THC, and DSF. They tended to present with a shorter duration of gait disturbance and fewer additional symptoms. Despite having the highest burden of vascular risk factors (hypertension 60%, cerebrovascular accident 33%, and hyperlipidemia 30%), they experienced a 78% response rate, with the fewest shunt setting changes required.

This reinforces the prognostic utility of DESH in predicting shunt responsiveness,^19^ supported by a recent meta-analysis.^20^ The pooled PPV of DESH was 80%, consistent with the 78% response rate in this cluster. However, DESH shows a low NPV, with a pooled estimate of 41%, suggesting that a substantial number of patients who may benefit from shunt surgery would be excluded if relying solely on this metric. Although cerebrospinal fluid (CSF) drainage is often considered gold standard for determining surgical eligibility in patients without DESH,^2^ despite a PPV of 92%, its NPV remains low at 37%.^6^

We suggest naming this group Hakim disease/syndrome, given it was highly dissimilar to other groups and presented with typical DESH and iNPH symptoms.^8^ These results suggest a highly reversible CSF-dynamic disturbance and emphasize early identification of DESH-positive patients, regardless of vascular burden present on imaging, should prompt timely CSF diversion.

### Cluster 2: Dilated Sylvian Fissure Hydrocephalus

Cluster 2 included 22.2% (n = 42) of patients and was predominantly male (97%) with a low symptom burden, more often presenting with only 1 or 2 features of the iNPH triad with a longer duration of gait disturbance compared with their DESH-positive counterparts. Imaging in this group demonstrated DSF without THC, low vascular burden, and enlarged head circumference. The large head circumference may point to a congenital etiology or younger onset, similar to cluster 4. Despite not meeting DESH criteria, these patients demonstrated sustained gait improvement and the lowest postoperative deterioration. Although they had the shortest follow-up, those who were followed-up demonstrated stability in symptoms over time.

Interestingly, this group shared DSF with cluster 1 but rarely exhibited other DESH features.

This suggests that isolated radiological features, particularly DSF, may still predict substantial benefit from shunting, especially in the presence of early or isolated gait impairment. This challenges the binary use of DESH as a prerequisite for surgical intervention and highlights the importance of a more nuanced approach to radiological assessment and patient phenotyping.^21-23^

### Cluster 3: Small Vessel Disease with Ventriculomegaly

This group constituted 13.9% (n = 26) of patients and was predominantly older women. They exhibited the second-highest vascular burden among all clusters and broadest callosal angles. Unlike Cluster 1, patients in this cluster did not demonstrate typical DESH imaging features and presented with a more heterogeneous symptom profile including headaches (27%), seizures (12%), and visual changes (4%).

Although meeting criteria for probable iNPH, these patients demonstrated a shorter duration of response, with a proportion experiencing progressive decline despite initial response and multiple valve adjustments. In some, higher settings were maintained due to falls risk and concern for SDH in the setting of deterioration; in others, patients failed to have a sustained response after shunt setting reductions, in contrast to other clusters that improved with valve setting lowering. Despite this, patients still maintained a median response duration of 15 months [7-30], where with proactive follow-up and valve optimization a substantial proportion still proceed to achieve improvement despite the initial stable clinical state or decline. Such a response would be clinically significant for these patients, although this effect is variable with some experiencing a longer sustained and others shorter duration of response.

This group may represent a distinct pathophysiological entity and overlapping phenotype with subcortical small vessel disease (i.e., Binswanger disease), where vascular pathology disrupts CSF dynamics, without the morphological features of DESH.^24^ Evidence remains mixed, with a recent meta-analysis describing a more conservative shunt response in the presence of white matter changes,^5^ with others supporting reversibility with shunting.^25^ Comorbidity optimization may be key to improving outcomes in this subgroup. Patients did experience an initial benefit from shunting, suggesting an element of reversibility and potentially preventative role in this cohort; however, microvascular injury may limit the extent of response.^26^

### Cluster 4: Marked Ventriculomegaly

Cluster 4 represented 34% (n = 64) and was the youngest cohort. They exhibited the largest ventricles and large head circumference alongside low comorbidity. Headache (17%) and preexisting seizures (3%) were also more common. Notably, these patients experienced substantial benefit across all 3 symptom domains and quality of life.

In our center, patients with marked ventriculomegaly are often commenced on higher valve settings to reduce SDH formation.^27,28^ However, in the absence of significant vascular comorbidity, the risk appeared low, and subsequent reductions in settings were well tolerated. These findings support the safety and efficacy of such an approach and suggest that proactive follow-up with valve adjustments may expedite clinical optimization in this cohort. Multiple successful valve adjustments may also cause a delay in the reporting of favorable outcomes and contribute to the prolonged follow-up observed.

This cohort aligns with the phenotypic presentation of late midlife hydrocephalus^8^ or adult-onset congenital hydrocephalus.^29^ These patients may represent a distinct pathophysiological subtype with compensated congenital hydrocephalus, who maintain function through most of their lives but become symptomatic later in life, presenting with typical Hakim Triad symptoms and headache. Many of these patients had known incidental ventriculomegaly for years.^30^ The presence of marked ventriculomegaly without features of THC or DSF may indicate a more reversible CSF dynamic disturbance, offering a compelling rationale for early intervention and proactive valve titration.

### Findings in Context

Very few studies have explored the natural clustering of iNPH symptoms^7^ or applied clustering to other hydrocephalic conditions.^31,32^ A recent review explored alternative classification methods for chronic adult hydrocephalus, including iNPH.^8^ To the best of our knowledge, no studies have assessed clustering of outcomes, imaging, and comorbidities in iNPH.

Mazza et al^7^ combined symptoms and demographics, identifying 2 distinct dimensions. Older populations with iNPH presented as a subgroup with more severe symptoms and poorer quality of life compared with younger, more educated counterparts. A similar contrast was observed in our study, where patients with small-vessel disease and ventriculomegaly represented older, more symptomatic patients compared with the younger marked ventriculomegaly cluster.

Tullberg et al^5^ similarly described a spectrum of presentations for chronic hydrocephalus in adults, varying with age. Those presenting at older age tended to experience the typical hakim triad of symptoms with younger counter parts more likely to experience a tetrad incorporating headaches alongside an enlarged head circumference. Both features of this younger cohort were observed in cluster 3. Such findings, however, were not unique, as cluster 2 also exhibited enlarged skull dimensions but without additional symptoms, whilst cluster 3 comprised older patients frequently presenting with symptoms outside of this triad, suggesting a more nuanced approach is needed. This highlights the heterogeneity of characteristics across clusters and the importance of considering multiple factors for phenotyping.

These clusters may represent different pathophysiology or stages of disease progression. Our results demonstrate patients commonly grouped as iNPH can be delineated into distinct syndromes with characteristic phenotypes.^7,8^ Future studies should validate these clustering models prospectively and compare performance against existing classification methods. Consideration of these groups should also be integrated database analyses and decision-making aids with prospective evaluation of effectiveness.

### Strengths and Limitations

This analysis presents a novel methodology for investigating iNPH subtypes. Bias was minimized by using unsupervised machine learning, focused on identifying patterns without priori assumptions. Owing to the statistical techniques used, data were limited to variables with complete entries, restricting the metrics that could be evaluated with retrospective analysis. The single-center nature and inclusion of only shunted patients limited the sample size and may limit the generalizability of the results and introduce selection bias. We recognize that patient comorbidities progress with age and can affect the reported duration of improvement, similarly selection bias may be considered where patients who deteriorate or experience ongoing symptoms require more intensive, longer term follow-up.^33^

## CONCLUSION

Patients with probable iNPH typically present as 1 of 4 distinct clinical syndromes: (1) DESH-positive, (2) dilated sylvian fissure hydrocephalus, (3) small vessel disease with ventriculomegaly, and (4) marked ventriculomegaly. Unsupervised machine learning applied to clinical and radiological data identified meaningful patient subtypes, supporting the concept that iNPH may represent multiple disease entities. Integration of these subgroups into clinical pathways could improve patient stratification and guide tailored management strategies. In particular, it facilitates targeted research and refinement of clinical trial designs for better characterization of these iNPH subgroups.
